# Waste-to-Resource: Heavy Metal Ions Adsorption from Aqueous Solutions Using Coal Fly Ash and Bone Charcoal

**DOI:** 10.3390/molecules31142515

**Published:** 2026-07-18

**Authors:** Eleonora Sočo, Andżelika Domoń, Dorota Papciak

**Affiliations:** 1 Department of Inorganic and Analytical Chemistry, Faculty of Chemistry, Rzeszow University of Technology, 35-029 Rzeszow, Poland; 2 Department of Water Purification and Protection, Faculty of Civil, Environmental Engineering and Architecture, Rzeszow University of Technology, 35-029 Rzeszow, Poland; dpapciak@prz.edu.pl

**Keywords:** coal fly ash, bone charcoal, lead, cadmium, heavy metals, adsorption, Langmuir isotherm, kinetics

## Abstract

Finding cost-effective and eco-friendly ways to remove toxic heavy metals from wastewater remains a critical challenge for industrial sustainability. This study presents a comparative performance matrix of coal fly ash (CFA) and bone charcoal (BC) for the high-capacity remediation of Cd(II) and Pb(II) ions. This work establishes a direct cross-matrix comparison between a heterogeneous aluminosilicate phase (CFA) and a uniform calcium-phosphate structure (BC) under identical systemic boundaries. SEM/EDS, FT-IR, and complementary TG/DTG/DTA screenings confirmed that distinct material-specific functional frameworks drive a predominantly physical mechanism governed by electrostatic and van der Waals interactions. Equilibrium data fitted the non-linear Langmuir model well (R^2^ > 0.99 at 20 °C). BC proved to be significantly more effective, achieving maximum sorption capacities (q_max_ of 397.55 mg/g for Pb(II) and 325.09 mg/g for Cd(II), outperforming CFA (118.22 and 105.59 mg/g, respectively). Sorption capacities decreased with temperature up to 80 °C, confirming the exothermic nature of the process, which was further substantiated by negative enthalpy values (∆H^0^ = −7.27 to −14.19 kJ/mol). Thermodynamic parameters indicated a spontaneous process (∆G^0^ < 0, −9.55 to −19.33 kJ/mol) with positive entropy changes (∆S^0^ = 5.82 to 39.09 J/(mol·K)). Adsorption kinetics followed the pseudo-second-order model, with intraparticle diffusion acting as a key rate-limiting step. Regardless of the adsorbent, Pb(II) ions were immobilized faster and more efficiently than Cd(II) due to a smaller hydration radius. In conclusion, both industrial by-products represent promising, sustainable options for heavy metal wastewater treatment, with BC demonstrating superior performance.

## 1. Introduction

With the dynamic increase in the volume of generated industrial solid waste, environmental pollution has become one of the most serious challenges of the modern world. The global energy sector, still largely based on coal extraction and combustion, generates millions of tons of coal fly ash (CFA) annually. The scale of this phenomenon is illustrated by environmental reports, according to which coal-fired power plants in India alone produce approximately 131 million tons of CFA per year, while in China, this volume has reached as much as 600 million tons [1,2]. The magnitude of the challenge is perfectly demonstrated by the fact that the global demand for coal reached its historical peak in 2025 at as much as 8.85 billion tons, thereby generating record amounts of combustion by-products [3]. Uncontrolled disposal of coal fly ash carries severe ecological and health risks. Fine solid particles of CFA can contribute to the formation of smog and haze, causing profound pollution of the atmospheric air, and their inhalation poses a direct threat to human health [4,5,6]. Furthermore, a critical environmental issue during the landfilling of CFA is the intensive leaching of toxic heavy metals contained within them into the soil and groundwater. Therefore, the rational management and disposal of this waste currently represents a significant economic and technological burden for many countries, forcing the search for alternative routes for its recycling [7,8,9,10,11].

To date, coal fly ash has found wide application in the construction industry, including as an additive (10–20%) in the production of cement and Portland concrete, demonstrating a positive effect on its durability, hardening kinetics, and final compressive strength [12,13]. Furthermore, CFA is successfully utilized as a precursor in the synthesis of zeolites and mesoporous silica, as well as an efficient adsorbent for capturing carbon dioxide, sulfur dioxide, nitrogen oxides, and metallic mercury from flue gases. The chemical composition of CFA, rich in mineral compounds such as silica, alumina, and iron oxides, also opens new perspectives in the synthesis of geopolymers designed, among other purposes, for electromagnetic interference shielding [1,2,14,15,16]. The physicochemical and structural properties of coal fly ash depend not only on the type of coal burned but also on the parameters of the technological process itself, including the boiler type (conventional or fluidized bed), temperature, and fuel particle size distribution [2,17,18].

Concurrently, the global meat industry generates millions of tons of bone waste annually. According to forecasts by the Organisation for Economic Co-operation and Development (OECD), a further increase in global meat production by another 40 million tons is anticipated over the next decade [19]. One of the most effective methods for the safe eradication of pathogens contained in animal raw materials is thermal treatment (to mitigate the risk of Creutzfeldt-Jakob disease, the skull and vertebral column are excluded) [20,21]. As a result of the pyrolysis of bone waste—primarily bovine and porcine—in sealed reactors at temperatures up to 700 °C, bone charcoal (BC) is obtained. It is a porous, black solid consisting of 70–76% hydroxyapatite (calcium hydroxyphosphate), 7–9% calcium carbonate, and 9–11% amorphous carbon [22,23]. The final structural properties of BC are determined by the origin of the raw material and the pyrolysis conditions (residence time, heating rate, type of purge gas, and temperature, which usually ranges between 350 and 900 °C) [24]. For years, due to its deep color, bone charcoal was utilized as a pigment (known as ivory black) in the textile and tanning industries, as well as in the production of polishing pastes [21]. It also found wide application in the sugar industry for decolorizing syrups during cane sugar refining, where it is currently being replaced by synthetic ion-exchange polymers and conventional activated carbons [23,25]. Nowadays, owing to the presence of the hydroxyapatite matrix, BC represents a highly promising and low-cost adsorbent dedicated to the removal of toxic heavy metal ions from wastewater and liquid industrial effluents [25].

The presence of heavy metals, such as lead, cadmium, nickel, copper, and zinc, constitutes one of the most severe threats to both aquatic and terrestrial ecosystems due to their high toxicity, bioaccumulation capacity, and persistence against biodegradation. Prolonged exposure of living organisms to these xenobiotics, even at extremely low concentrations, leads to severe organ damage (including the kidneys, liver, brain, and lungs), as well as mutagenic effects that can result in infertility or spontaneous abortions [26,27]. Chronic exposure to cadmium is particularly hazardous, resulting in renal tubular dysfunction, proteinuria, alveolar damage, and the induction of carcinogenic processes. In clinical diagnostics, the concentration of this element in whole blood is established as the criterion for toxicity; for adults without occupational exposure, the permissible reference value is below 5 μg/L [28]. Lead exerts an equally destructive impact on human and animal health. In plants, exposure to Pb impairs the photosynthetic apparatus and inhibits growth, which facilitates its penetration into higher trophic levels of the food chain [29,30]. Chronic lead intoxication (lead poisoning) induces irreversible neurodegenerative changes (especially in children, impairing intellectual development), gastrointestinal disorders, muscle weakness, and a decline in semen quality. Medical intervention in cases of acute lead poisoning relies on chelation therapy utilizing dimercaprol, 2,3-dimercaptosuccinic acid (DMSA), or ethylenediaminetetraacetic acid (EDTA) salts [29,31]. The lead level in the blood of an adult human should not exceed the reference value of 0.035 mg/L (35 μg/L), which is also applicable to children [32,33].

Among the wide range of physicochemical methods used to remove heavy metal ions from wastewater, adsorption is considered the most efficient, versatile, simple to use, and economically viable. However, although both CFA and BC have been independently studied in the past, the existing literature lacks direct comparisons of both materials that contrast their structural behavior under identical experimental and thermodynamic conditions with high metal ion concentrations. Therefore, this study assessed the application potential of these two different material structures in their raw, unmodified forms. Using these industrial byproducts, this work focuses on waste reuse, which directly aligns with the fundamental principles of the circular economy. The novelty of this work is based on the integration of macroscopic adsorption tests with advanced instrumental surface characterization. The use of precise SEM-EDS elemental mapping, determination of the point of zero charge (pH_PZC_), and multi-stage thermogravimetric and differential thermodynamic profiles (TG/DTG/DTA) allowed for a structural comparison of both adsorbents. The study included a multifaceted analysis of adsorption equilibria using the classical, nonlinear Langmuir isotherm model. Furthermore, the kinetic and thermodynamic parameters of the process were verified based on selected mathematical models, which, combined with spectroscopic and thermal data, allowed for a precise determination of the adsorption mechanism.

## 2. Results

### 2.1. Characterization of Adsorbents

#### 2.1.1. Structural, Morphological, and Surface Charge Analysis (SEM-EDS and pH_PZC_)

**Figure 1:**
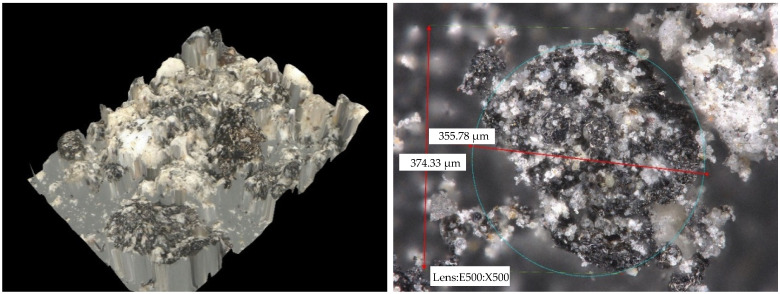
Microscopic view of coal fly ash (500× magnification).

**Figure 2:**
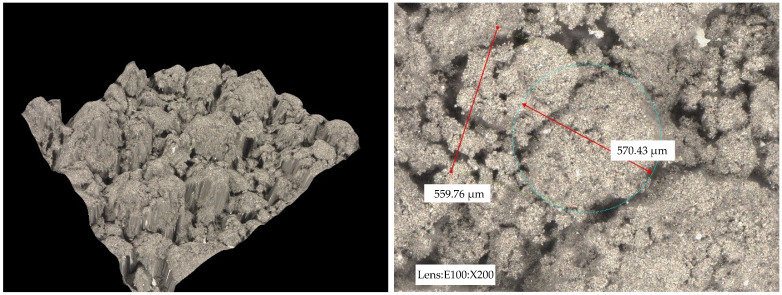
Microscopic view of bone charcoal (500× magnification).

To evaluate the application potential of coal fly ash and bone charcoal for the removal of cadmium and lead, their structural and morphological properties were analyzed in the first stage of the study. Digital optical microscopy analysis performed with a Keyence microscope allowed for the determination of the grain sizes, which were found to be approximately 356 × 374 μm for coal fly ash (Figure 1) and 570 × 560 μm for bone charcoal (Figure 2). Surface morphology analysis revealed that the bone charcoal surface is more uniform, with grains exhibiting a tendency to form larger clusters, i.e., agglomerates. In contrast, the individual grains of coal fly ash present a distinct morphology, allowing for easy morphological classification. Furthermore, it is noteworthy that the surface texture of bone charcoal becomes increasingly rough and porous when the pyrolysis process is conducted at higher temperatures and for extended durations [23].

**Figure 3:**
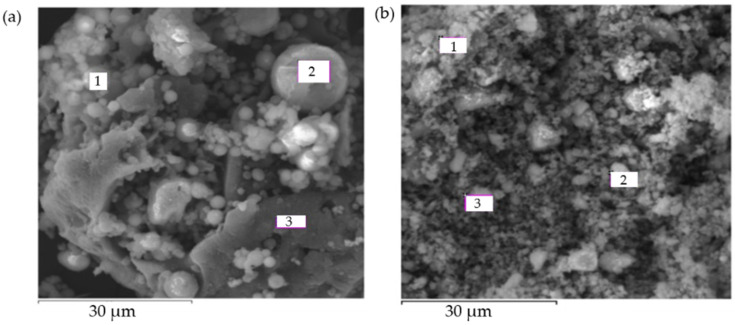
SEM micrograph showing the surface morphology with marked analysis areas of: (**a**) coal fly ash; (**b**) bone charcoal (points 1–3 indicate EDS microanalysis areas).

The elemental composition, determined via EDS point analysis, also exhibited significant differences between the two samples. For the coal fly ash (Figure 3a, Table 1), a dominance of oxygen and carbon was observed, along with aluminum and silicon, which are typical constituents of aluminosilicates. Individual measurement points for this material revealed substantial variations in the local chemical composition (e.g., a high carbon content at point 3), directly confirming the optical microscopy findings regarding the inherent heterogeneity of this material. In contrast, the bone charcoal matrix (Figure 3b, Table 2) was characterized by high chemical homogeneity across all analyzed points, as evidenced by very low standard deviations (±SD). This result corresponds perfectly with the earlier observation of the uniform morphology of the material. Bone charcoal features an exceptionally high content of calcium and phosphorus, unambiguously confirming its hydroxyapatite-based structure.

**Figure 4:**
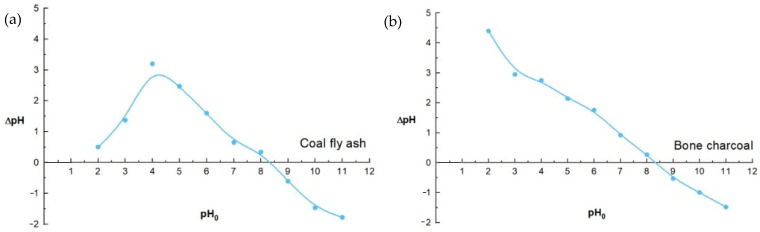
Plot of pH change (∆pH) as a function of initial pH for: (**a**) coal fly ash, and (**b**) bone charcoal.

Another highly important parameter determining the surface properties of the studied materials, which is directly linked to their mineral composition, is the point of zero charge (PZC). It is defined as the pH value of a solution at which the net surface charge of the solid suspension is equal to zero. This condition is met when the charges of cations and anions present on the adsorbent surface mutually balance each other. At the pH_PZC_ point, the solid material is characterized by its maximum hydrophobicity, hardness, and sedimentation rate. This value depends directly on the interactions of OH^−^ and H^+^ ions present in the solution with the functional groups on the adsorbent surface. If the pH of the studied system is lower than the pH_PZC_ value, the adsorbent surface undergoes protonation, acquiring a positive charge, which favors the binding of anions. Conversely, when the pH of the system is higher than the pH_PZC_ value, the surface undergoes deprotonation and accumulates a net negative charge, thereby favoring the adsorption of cations [34]. In the case of the analyzed coal fly ash and bone charcoal, similar pH_PZC_ values were obtained, amounting to 8.3 and 8.4, respectively (Figure 4).

#### 2.1.2. Thermogravimetric and Differential Thermal Analysis (TG/DTG-DTA)

**Figure 5:**
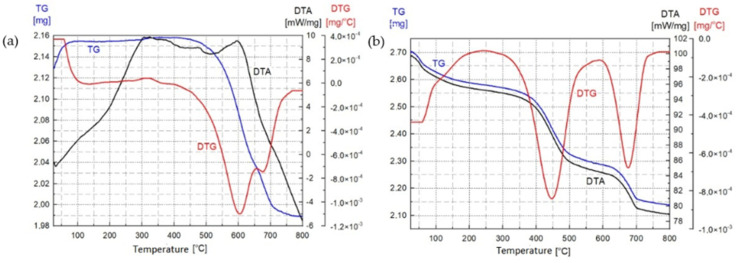
TG, DTG, and DTA thermograms of the investigated adsorbents: (**a**) coal fly ash, and (**b**) bone charcoal.

The thermal characterization of the investigated adsorbents within the temperature range of 30–800 °C is presented in Figure 5, and their key thermal degradation parameters are comprehensively summarized in Table 3. Interestingly, within the initial temperature range from 30 °C to approximately 320 °C, the TG curve exhibits a slight mass increase (from approximately 2.13 mg to 2.16 mg). This phenomenon, accompanied by a steady increase in the DTA signal, is attributed to the initial oxidation of unburned carbon or specific mineral phases present in the ash, which counteracts and dominates over any potential moisture loss (Figure 5a). Above 500 °C, the material undergoes a major thermal degradation stage, initiated at T_onset_ = 550 °C. The derivative thermogravimetric (DTG) curve in this region highlights two overlapping mass loss steps: the first acceleration corresponds to a broad, intense exothermic envelope on the DTA curve peaking near 600 °C (T_max1_), which represents the intensive oxidation and burning of residual carbonaceous matter. This is immediately followed by a second DTG acceleration peaking at 675 °C (T_max2_), overlapping with the T_5%_ weight loss threshold. This second step is associated with the endothermic decomposition of carbonate minerals (decarbonation of CaCO_3_) and structural changes within the aluminosilicate matrix, causing the sharp decline of the DTA curve above 680 °C. Due to the initial oxidation and high thermal stability of the inorganic matrix, the coal fly ash does not reach 10% or 50% mass loss thresholds within the tested range, displaying an exceptionally high residual mass of 98.1% at 600 °C (Table 3).

A fundamentally different thermal behavior is observed for the bone charcoal sample (Figure 5b). The TG and DTA profiles in the range of 25–250 °C reflect a conventional endothermic dehydration process, where the evaporation of physically bound water drives the material past its T_5%_ threshold at 230 °C (Table 3). The primary degradation stage accelerates significantly after T_onset_ = 350 °C, leading to a sharp DTG mass loss peak centered at 450 °C (T_max1_). This stage is driven by the pyrolytic destruction and decomposition of residual organic matter embedded within the bone structure, closely matching the T_10%_ value of 445 °C. In the subsequent interval (450–600 °C), the decomposition rate slows down as the carbonaceous residues finish volatilizing. Upon reaching higher temperatures (650–800 °C), a final, distinct endothermic reaction is registered on the DTA curve, perfectly corresponding to a deep DTG peak at 680 °C (T_max2_). This high-temperature stage represents the decarbonation of surface-bound carbonates alongside the structural restructuring and partial sintered destruction of the crystalline hydroxyapatite mineral core.

When comparing both materials based on the parameters compiled in Table 3, it is evident that the coal fly ash possesses significantly higher overall thermal stability than the bone charcoal. This is reflected by the much higher T_5%_ (°C) value (675 °C vs. 230 °C), the significantly delayed onset of active weight loss (T_onset_ of 550 °C vs. 350 °C), and a substantially larger residual mass at 600 °C (98.1% vs. 84.1%).

#### 2.1.3. Surface Functional Groups Identification (FTIR Spectroscopy)

**Figure 6:**
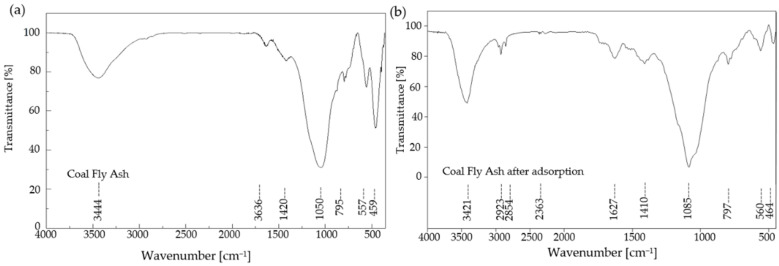
FT-IR spectra of coal fly ash: (**a**) before adsorption, and (**b**) after the adsorption of Cd(II) and Pb(II) ions.

**Figure 7:**
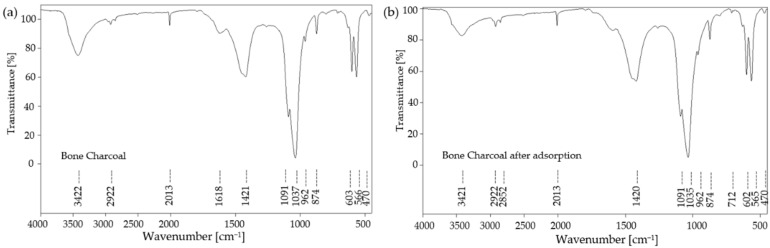
FT-IR spectra of bone charcoal: (**a**) before adsorption, and (**b**) after the adsorption of Cd(II) and Pb(II) ions.

Using Fourier-transform infrared (FT-IR) spectroscopy, the spectra for the investigated adsorbent samples were recorded before and after the adsorption of cadmium(II) and lead(II) ions (Figure 6 and Figure 7). The detailed assignments and interpretations of the recorded absorption bands are summarized in Table 4. The spectroscopic analysis revealed that the overall spectral profile for the initial materials and those after the adsorption process remains similar; however, distinct differences are noticeable in the intensity and the exact position of individual absorption bands. In the spectra of the coal fly ash (Figure 6), the presence of characteristic bands was determined around the wavenumbers of: 3444 cm^−1^, 1636 cm^−1^, 1420 cm^−1^, 1050 cm^−1^, and 795 cm^−1^. The broad band in the 3400–3450 cm^−1^ range is induced by the stretching vibrations of hydroxyl (OH) groups involved in the formation of intermolecular hydrogen bonds on the adsorbent surface [35,36]. The subsequent band at approx. 1636 cm^−1^ corresponds to the bending vibrations of bound water molecules and the stretching vibrations of C=C bonds in aromatic systems, activated by the presence of neighboring oxygen atoms within the ash structure [37]. A weak signal in the 1600–1800 cm^−1^ region can be assigned to the stretching vibrations of carbonyl groups (C=O) of organic origin (derived from aldehydes and ketones). The band at the wavenumber of 1420 cm^−1^ corresponds to the stretching vibrations of C–O bonds contained within carboxylic or ether groups. The most intense and broad band, with a maximum at approx. 1050 cm^−1^, constitutes a key indicator of the ash mineral phase and is generated by the asymmetric stretching vibrations of Si–O–Si(Al) bonds. The final adsorption band in the 795 cm^−1^ region corresponds to the asymmetric stretching vibrations of SiO_4_^4−^ and AlO_4_^5−^ bonds, which are characteristic of the aluminosilicates contained in the coal fly ash [38,39]. Furthermore, low-intensity and narrow bands are present in the FT-IR spectrum within the fingerprint region of ῡ = 459–557 cm^−1^, relating to the bending vibrations within the Si–O and Al–O bond systems. A comparison of the fly ash spectrum before adsorption (Figure 6a) and after the adsorption of Cd(II) and Pb(II) ions (Figure 6b) reveals a distinct shift in the main maximum of the aluminosilicate matrix from 1050 cm^−1^ to 1085 cm^−1^, as well as an alteration in the shape and a shift in the peak representing OH groups (to the value of 3421 cm^−1^). Moreover, after the adsorption process, new bands appeared in the ash spectrum at wave numbers 2923 cm^−1^ and 2854 cm^−1^, characteristic of stretching vibrations of aliphatic CH_2_ and CH_3_ groups. The appearance of these signals was probably related to trace organic residues introduced during sample preparation (e.g., matrix effect). Therefore, these specific aliphatic bands cannot be directly related to chemical interactions between the active sites of the adsorbent and heavy metal cations.

The FT-IR spectra of the bone charcoal sample before and after the adsorption of Cd(II) and Pb(II) ions (Figure 7) exhibit a similar pattern of absorption bands, confirming the stability of the matrix’s mineral skeleton; however, they differ in terms of signal intensity and width. Key characteristic bands for the hydroxyapatite structure, which constitutes the main component of bone charcoal, were identified at approximately: 3422 cm^−1^, 2013 cm^−1^, 1420 cm^−1^, 1035 cm^−1^, and 874 cm^−1^. The broad band with a maximum at 3422 cm^−1^ corresponds to the stretching vibrations of hydroxyl (OH) groups involved in hydrogen bonding. The band around 2013 cm^−1^ represents a combination (harmonic) band of the phosphate matrix lattice vibrations. A distinct signal at 1420 cm^−1^ and a smaller peak at 874 cm^−1^ are directly related to the stretching and bending vibrations, respectively, of carbonate groups (CO_3_^2−^) incorporated into the crystalline structure of hydroxyapatite. The dominant, most intense band with a maximum at 1035 cm^−1^ (with a visible shoulder at 1090 cm^−1^) originates from the asymmetric stretching vibrations of phosphate groups (PO_4_^3−^). In turn, within the fingerprint region of 565–602 cm^−1^, a sharp, well-resolved doublet of bands is recorded, which corresponds to the internal bending vibrations of O–P–O bonds in the phosphate tetrahedra. A comparison of both spectra (Figure 7a,b) indicates that the heavy metal ion adsorption process induced changes in the intensity of the bands representing phosphate groups (1035 cm^−1^ and 565 cm^−1^) and carbonate groups (1420 cm^−1^). Furthermore, a new band appeared at 712 cm^−1^ in the spectrum of bone charcoal after adsorption, accompanied by an increase in intensity and a clearer resolution of the peaks in the 2852–2922 cm^−1^ region originating from aliphatic group vibrations. The appearance of these signals was probably related to trace organic residues introduced during sample preparation.

### 2.2. Adsorption Studies

#### 2.2.1. Evaluation of Adsorption Capacity: Effect of pH and Isotherm Studies

**Figure 8:**
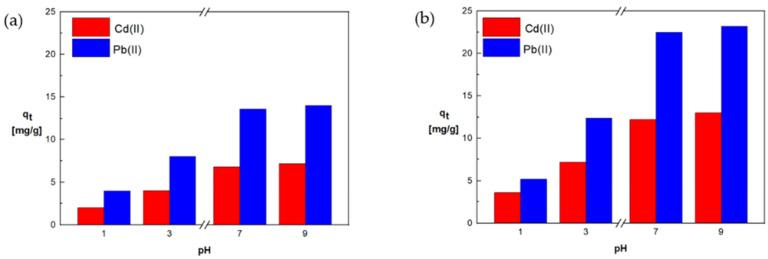
Effect of initial pH on the adsorption capacity of: (**a**) coal fly ash and (**b**) bone charcoal for Cd(II) and Pb(II) ions (initial concentration: 600 mg/L).

Bone charcoal exhibits a significantly higher adsorption capacity for Pb(II) ions than coal fly ash, reaching a maximum adsorption capacity of approximately 22.5–23.1 mg/g in an environment with pH = 7–9 (Figure 8b). In a strongly acidic environment (pH = 1–3), the efficiency of the process for both investigated cations is distinctly lower. This directly results from the fact that at such low pH values, the adsorbent surface carries a strong positive charge (pH < pH_PZC_), which generates electrostatic repulsion between the active centers and the Pb(II) and Cd(II) cations. Additionally, the metal ions compete for the same active sites with the highly abundant H^+^ ions. In the case of coal fly ash, a similar increasing trend is observed—the adsorption of lead(II) ions dominates at higher pH values (7 and 9). However, the maximum amount of adsorbed Pb(II) ions is lower than that for the bone charcoal, amounting to approximately 13.8 mg/g (Figure 8a). For both analyzed adsorbents, it was noted that regardless of the pH of the medium, the binding process of Pb(II) ions proceeds with greater efficiency than that of Cd(II) ions. This behavior can be attributed to the smaller hydration radius of lead and its higher chemical affinity toward the mineral structures of the investigated adsorbents.

**Figure 9:**
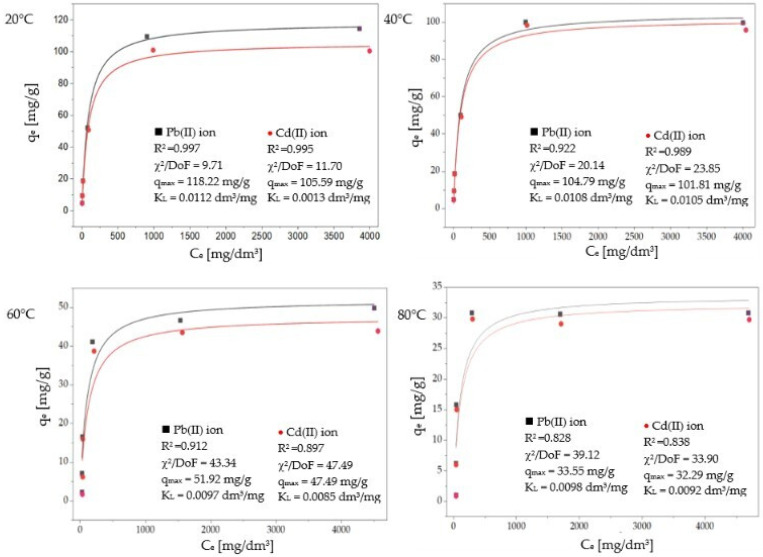
Langmuir adsorption isotherms of Pb(II) and Cd(II) ions for coal fly ash at temperatures of 20, 40, 60, and 80 °C.

**Figure 10:**
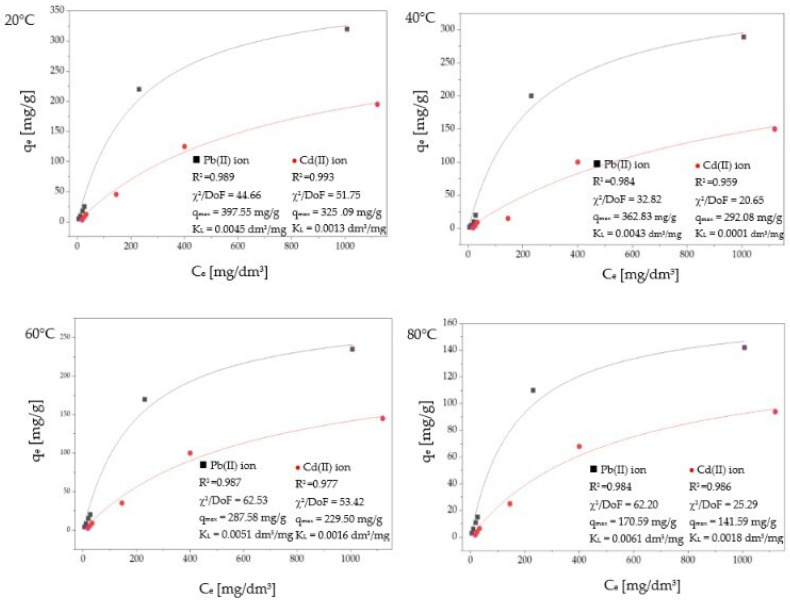
Langmuir adsorption isotherms of lead(II) and cadmium(II) ions for bone charcoal at temperatures of 20, 40, 60, and 80 °C.

**Figure 11:**
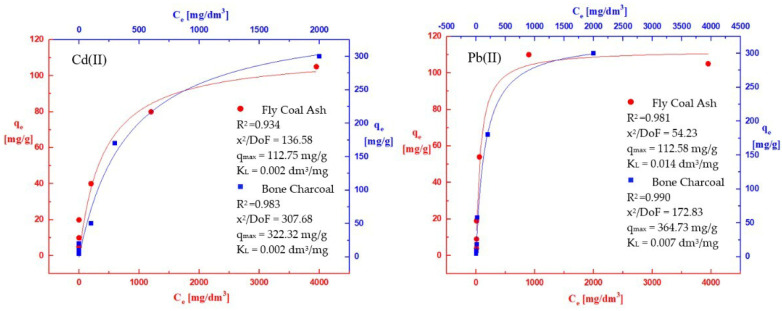
Langmuir adsorption isotherms of Cd(II) and Pb(II) ions as a function of their concentration for coal fly ash and bone charcoal.

The isotherms generated using OriginPro software (Figure 9 and Figure 10) present the dependence of the equilibrium adsorption capacity (q_e_) on the equilibrium concentration of the investigated metal ions in the aqueous phase (C_e_) across four temperature variants: 20 °C, 40 °C, 60 °C, and 80 °C. Statistical analysis revealed that the experimental data showed the highest goodness-of-fit to the theoretical Langmuir model at the lowest tested temperature of 20 °C. The coefficients of determination (R^2^) for Pb(II) and Cd(II) where 0.997 and 0.995 for coal fly ash, and 0.989 and 0.993 for bone charcoal, respectively. As the system temperature increased, a successive decrease in the R^2^ values was observed, indicating a slight reduction in the adequacy of the adopted model at higher temperature ranges. A crucial parameter determined from the Langmuir equation is the maximum adsorption capacity (q_max_), which defines the saturation limit of the adsorbent monolayer. The highest values of this parameter were recorded at 20 °C for both the coal fly ash (q_max_ = 118.22 mg/g) and the bone charcoal (q_max_ = 397.55 mg/g). A comparison of both adsorbents at this temperature indicates that bone charcoal is characterized by a nearly four-fold higher binding capacity for the investigated cations per unit mass of the adsorbent. The observed distinct decrease in q_max_ values with increasing temperature (a reduction in capacity to 33.55 mg/g for the ash and 170.59 mg/g for the bone charcoal at 80 °C for Pb(II)) explicitly indicates the exothermic nature of the analyzed adsorption process. This suggests that supplying thermal energy to the system promotes ion desorption or weakens the electrostatic and chemical interactions between the surface active centers and the adsorbed cations. A direct comparison of the adsorption efficiency of both investigated materials toward individual cations is presented in Figure 11. The superior efficiency of bone charcoal compared to coal fly ash with respect to both cations directly results from its more developed porous structure and the presence of specific phosphate and carbonate groups that exhibit a high affinity toward heavy metal species through surface complexation and outer-sphere electrostatic interactions [44]. The structural hydroxyapatite skeleton of bone charcoal provides stable, well-distributed active locations that can easily accommodate Pb(II) [45]. To better illustrate the advantages and limitations of the materials investigated in this work, a comparative analysis of their maximum adsorption capacities against previously published data for different raw and modified adsorbents is summarized in Table 5.

It is important to note that since the adsorption experiments were conducted in a binary system containing both Cd(II) and Pb(II) ions simultaneously, competitive adsorption for the available active sites on the adsorbents took place. While the single-component Langmuir model serves as a valuable tool to determine the apparent maximum adsorption capacities under these conditions, the clear dominance of Pb(II) over Cd(II)—manifested by its significantly higher q_max_ and faster equilibration times—highlights the competitive advantage of lead ions. This behavior is strongly governed by the physical–chemical properties of the cations. Pb(II) ions possess a smaller hydrated ionic radius (4.01 Å) compared to Cd(II) (4.26 Å), allowing them to diffuse more rapidly into the porous networks of both coal fly ash and bone charcoal. Furthermore, it should be emphasized that under real-world conditions, wastewater may also contain other competing ions (e.g., Ni(II), Zn(II), SO_4_^2−^) and natural organic matter (NOM) that will compete for the active sites on the adsorbent surfaces [46,47]. Moreover, while this study successfully establishes the high baseline binding efficiency of both materials, the recovery and recycling of the immobilized Pb(II) and Cd(II) ions remain essential considerations for sustainable wastewater management. Structural and chemical regeneration of the spent adsorbents, along with metal stripping optimization, represents a distinct technological challenge. Depending on the adsorbent matrix, desorption and resource recovery can be conducted via chemical methods using mineral or organic acids (e.g., HCl, HNO_3_, H_2_SO_4_), alkaline solutions, or salt eluents (NaCl, KCl). Additionally, advanced thermal, microwave-assisted, electrochemical, or ultrasonic regeneration techniques can be deployed to restore active surface sites [48,49,50,51].

**Table 5:** Comparative analysis of maximum monolayer adsorption capacities (q_max_) for Pb(II) and Cd(II) ions using various similar adsorbents at room temperature.

Adsorbent	Metal	q_max_ (mg/g)	References
Coal fly ash	Pb(II)	112.58	This study
	Cd(II)	112.75	
Bone charcoal	Pb(II)	364.73	This study
	Cd(II)	322.32	
Pure zeolite	Pb(II)	432.57	[52]
Maize stalk biochar	Pb(II)	62.86	[53]
White pottery clay	Pb(II)	155.04	[54]
	Cd(II)	26.991	
Rice husk biochar	Pb(II)	37.20	[55]
	Cd(II)	9.22	
Manure biochar	Pb(II)	175.53	[56]
	Cd(II)	68.08	
Bone Char (BC) Microcrystalline Cellulose-Bone Char	Pb(II)	89.9 115.7	[57]
Cow bones	Pb(II)	0.133	[58]

#### 2.2.2. Adsorption Thermodynamics

**Figure 12:**
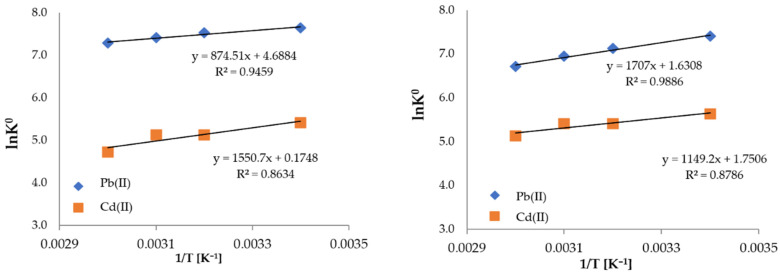
The relationship lnK_L_ = f(1/T) for the adsorption process of lead(II) and cadmium(II) ions using coal fly ash and bone charcoal.

Based on the slope of the straight line, the thermodynamic parameters of the process were determined (Figure 12), including the Gibbs free energy change (ΔG^0^), enthalpy change (ΔH^0^), and entropy change (ΔS^0^) (Table 6). Data analysis indicates that in both studied cases—i.e., for coal fly ash and bone charcoal—the enthalpy change value is negative. For coal fly ash, it amounts to −7.27 and −12.89 kJ/mol, respectively, while for bone charcoal, it is −9.55 and −14.19 kJ/mol, respectively. This unambiguously confirms the exothermic nature of the adsorption process of cadmium(II) and lead(II) ions onto the analyzed materials. The obtained ΔH^0^ values for both adsorbents fall strictly within the energy range typical for physical adsorption, which is generally considered to be below 40 kJ/mol [59,60]. The entropy change (ΔS^0^), which serves as a measure of disorder at the phase boundary, takes positive values (ΔS^0^ > 0) within the range of 5.82÷39.07 J/(mol·K) for the analyzed adsorbents. This demonstrates a high affinity between the adsorbate and adsorbent, as well as an increase in the degrees of freedom of the system at the phase boundary during the process (which typically results from the dehydration of metal ions prior to their binding onto the surface).In the analyzed system, the Gibbs free energy change values are negative (ΔG^0^ < 0), indicating that the adsorption of Cd(II) and Pb(II) ions is a spontaneous process. The fact that these values remain above the −20 kJ/mol threshold (i.e., closer to zero) confirms the predominant contribution of physical interactions (physisorption). The presented thermodynamic parameters confirm the significant influence of temperature on the course of the process. Since the process is exothermic, the efficiency of the adsorption of the investigated ions decreases with increasing temperature, as evidenced by a decrease in the equilibrium constant (lnK^0^). The high values of the coefficients of determination (R^2^) confirm an excellent fit of the experimental data to the applied linear model.

#### 2.2.3. Adsorption Kinetics and Contact Time Performance

**Figure 13:**
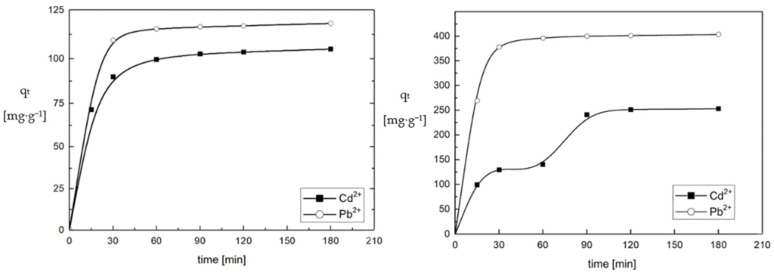
Effect of contact time on the adsorption of Cd(II) and Pb(II) ions onto coal fly ash and bone charcoal (experimental points represent mean values; *n* =3).

The adsorption of Cd(II) ions and Pb(II) onto coal fly ash reached equilibrium at 90 min with a capacity of q_t_ = 100, whereas the equilibrium for Pb(II) ions was established at 180 min with a capacity of q_t_ = 118 mg/g (Figure 13). For bone charcoal, the adsorption equilibrium for cadmium(II) ions was established at 120 min, yielding a q_t_ value of approximately 250 mg/g. Notably, the adsorption kinetics of lead(II) ions onto bone charcoal were remarkably rapid; the saturation of the adsorbent surface at approximately 400 mg/g was achieved within the first 30 min of the process, maintaining equilibrium up to 180 min. The experimental points in Figure 13 represent the mean values of three independent replicates (*n* = 3). It is worth noting that the associated measurement uncertainty (Student’s t-distribution confidence intervals at *p* = 0.05) was exceptionally small (<2.5%) and fell within the margins of the graphical symbols used, thereby making the error bars visually fully obscured by the data markers. Due to the superior adsorption performance and higher efficiency exhibited by bone charcoal compared to coal fly ash, the subsequent kinetic modeling was exclusively performed for this adsorbent.

**Figure 14:**
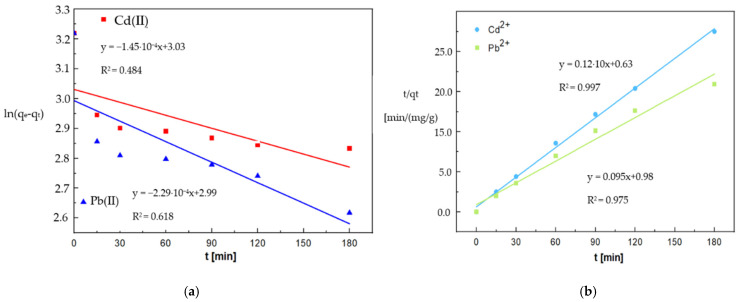
Linearized kinetic plots for the adsorption of cadmium(II) and lead(II) ions onto bone charcoal: (**a**) pseudo-first-order (PFO) model, (**b**) pseudo-second-order (PSO) model.

**Figure 15:**
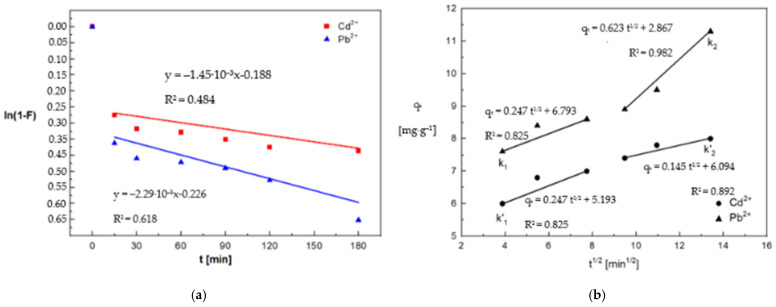
Linearized diffusion models for the adsorption of cadmium(II) and lead(II) ions onto bone charcoal: (**a**) film diffusion model, (**b**) Weber–Morris intraparticle diffusion model.

**Figure 16:**
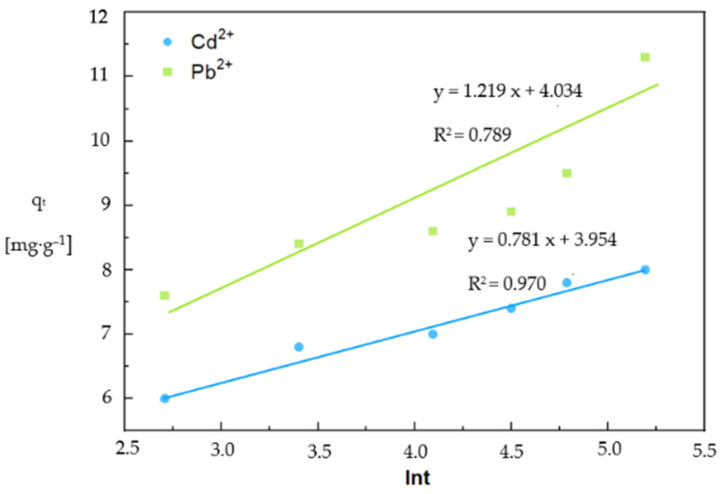
Linearized Elovich kinetic plots for the adsorption of cadmium(II) and lead(II) ions onto bone charcoal.

To comprehensively evaluate the adsorption mechanism and rate parameters of cadmium(II) and lead(II) ions onto bone charcoal, the experimental data were subjected to kinetic modeling. Five kinetic equations were applied: the pseudo-first-order (PFO), pseudo-second-order (PSO), film diffusion, intraparticle diffusion (Weber–Morris), and Elovich models (Figure 14, Figure 15 and Figure 16, Table 7).

Based on the parameters summarized in Table 7, the pseudo-second-order (PSO) kinetic model provided the best fit for the adsorption of cadmium(II) and lead(II) ions onto bone charcoal. This was evidenced by the highest coefficients of determination, which reached R^2^ = 0.997 for Cd(II) ions and 0.975 for Pb(II) ions. In comparison, the experimental data did not adequately fit the pseudo-first-order (PFO) model, yielding significantly lower R^2^ values (R^2^ < 0.618). The obtained kinetic parameters, particularly the PSO model fit and k_2_ values, are strictly linked to the high initial concentration (600 mg/L). Theoretically, variations in the initial adsorbate concentration can significantly alter the kinetic rate constants (k_1_, k_2_). As demonstrated in similar heavy metal systems, an increase in the initial concentration accelerates the collision frequency between metal ions and active sites while sharpening the concentration gradient. This strong thermodynamic driving force can shift the calculated rate constants toward higher values alongside elevated equilibrium capacities (q_e_) [61]. Although conducting the kinetic study at a single concentration represents a limitation, it successfully allowed for evaluating the maximum kinetic performance of bone charcoal under heavy pollutant loading and clearly identifying the overall kinetic mechanism of the process.

Based on the linear determination coefficients, the applicability of the kinetic models for cadmium(II) ions decreased in the following order: pseudo-second-order > Elovich > intraparticle diffusion > film diffusion/pseudo-first-order. For lead(II) ions, the sequence was established as: pseudo-second-order > intraparticle diffusion > Elovich > film diffusion > pseudo-first-order. According to the multi-linear plots of the Weber–Morris intraparticle diffusion model (Figure 15), the adsorption process for both metal ions occurs via two distinct stages. The first, rapid stage is attributed to external boundary layer (film) diffusion and mass transfer to the easily accessible active sites on the bone charcoal surface. The second stage, characterized by a lower slope, represents the actual intraparticle diffusion within the porous structure of the adsorbent, acting as the rate-limiting step. Since the regression lines do not pass through the origin, intraparticle diffusion is not the sole rate-controlling mechanism. The b coefficient in this model denotes the thickness of the boundary layer; the higher the value, the greater the contribution of the boundary layer to the adsorption process. In the Elovich model, high values of the linear coefficient of determination for cadmium(II) ions (R^2^ = 0.970) can also be observed, suggesting the predominantly physical nature of the adsorption process and reflecting the high energetic heterogeneity of the bone charcoal surface.

## 3. Materials and Methods

### 3.1. Materials

Two adsorbents were used in the adsorption studies: (1) coal fly ash obtained from a combined heat and power plant, and (2) bone charcoal purchased from Sigma-Aldrich. A bulk sample of the coal fly ash, weighing approximately 5 kg, was dried at room temperature (20 ± 1 °C) for two weeks. Subsequently, the bulk sample was reduced using the quartering method to obtain a laboratory sample of approximately 1 kg. The final preparation step involved sieving the laboratory sample to a grain size fraction of d ≤ 0.6 mm. A comparison of the physicochemical parameters and characteristics of both adsorbent materials is summarized in Table 8.

### 3.2. Characterization of Adsorbents

#### 3.2.1. Microscopic and Morphological Analysis

The microscopic analysis of the studied adsorbents was performed using a digital microscope (VHX-7000 series, Keyence, Mechelen, Belgium). Morphological and textural observations of the surface were carried out using a scanning electron microscope (SEM) (TESCAN VEGA 3, Brno, Czech Republic) operating under high vacuum conditions. Prior to imaging, the samples were sputter-coated with a thin layer of silver to ensure adequate electrical conductivity. The SEM was additionally equipped with a back-scattered electron (BSE) detector (INCA x-act, Oxford Instruments, High Wycombe, UK) to expand the scope of the elemental composition analysis.

#### 3.2.2. Thermogravimetric Analysis (TGA)

Thermogravimetric analysis (TG/DTG/DTA) of the adsorbent samples was performed using a STARe system analyzer (Mettler-Toledo, Greifensee, Switzerland). The samples were placed in platinum crucibles and heated from 25 °C to 800 °C at a constant heating rate of 10 °C/min under a dynamic air atmosphere with a continuous flow rate of 20 mL/min. During the measurement, changes in the sample mass were recorded to yield the thermogravimetric (TG) and derivative thermogravimetric (DTG) curves, while differential thermal analysis (DTA) was simultaneously recorded to monitor thermal effects.

#### 3.2.3. Determination of the Point of Zero Charge (PZC)

To determine the point of zero charge using the suspension method, 0.5 g of the selected adsorbent (either coal fly ash or bone charcoal) was weighed into ten 100 cm^3^ beakers. Subsequently, 50 cm^3^ of a 0.1 M sodium nitrate solution was added to each beaker. In the next step, the initial pH values of the samples were adjusted to 2, 3, 4, 5, 6, 7, 8, 9, 10, and 11 using a pH meter, utilizing 0.1 M and 1 M solutions of nitric (V) acid and sodium hydroxide. The samples were then left for 24 h, after which the final pH in each beaker was remeasured. The point of zero charge was determined by plotting the change in pH as a function of the initial pH, i.e., ΔpH = f(pH_initial_).

#### 3.2.4. Fourier-Transform Infrared Spectroscopy (FT-IR)

Fourier-transform infrared (FT-IR) spectroscopy was employed to identify the functional groups responsible for heavy metal binding on the adsorbent surfaces. The spectra of the pristine materials (raw coal fly ash and bone charcoal) and the metal-laden adsorbents (after the adsorption of cadmium(II) and lead(II) ions at an initial concentration of 600 mg/L) were recorded using a Bruker spectrometer (Bruker, Billerica, MA, USA). The measurements were performed using KBr discs over the wavenumber range of 400–4000 cm^−1^ with a spectral resolution of 0.7 cm^−1^.

### 3.3. Determination of Cadmium(II) and Lead(II) Ions

The concentration of Cd(II) and Pb(II) ions in the solutions after the adsorption process was determined using flame atomic absorption spectrometry (F-AAS) with a PERKIN ELMER 3100 spectrometer. The measurements were performed at wavelengths optimized for each ion, i.e., 228.8 nm for cadmium(II) and 217.0 nm for lead(II), both with a slit width of 0.7 nm. Other operating parameters of the device were adjusted according to the manufacturer’s recommendations. Hollow cathode lamps dedicated to cadmium(II) and lead(II) ions were used as the radiation sources. Quantitative analysis was carried out using an air-acetylene flame. According to the instrument specification and the linearity ranges for the analyzed elements, calibration curves were prepared using standard solutions of cadmium(II) and lead(II) ions at concentrations of 1.0, 1.5, and 2.0 mg/L, and 9.0 and 20.0 mg/L, respectively. To record the calibration curves, the standard solutions of Cd(II) and Pb(II) ions were introduced into the burner flame via a capillary. Following the calibration, the experimental samples were analyzed under identical conditions. Samples exceeding the analytical range of the calibration curve were appropriately diluted.

### 3.4. Adsorption Studies

#### 3.4.1. Effect of pH on the Adsorption Process

To determine the effect of pH on adsorption efficiency, 0.5 g of the selected adsorbent (either coal fly ash or bone charcoal) was weighed into 100 cm^3^ Erlenmeyer flasks, followed by the addition of 50 cm^3^ of a binary solution containing both cadmium(II) and lead(II) ions, each at a concentration of 600 mg/L. Using a pH meter and HNO_3_ and NaOH solutions (at concentrations of 1 M and 0.1 M), the pH of the suspensions was adjusted to 1, 3, 7, and 9. The mixtures were shaken for 2 h at room temperature. Afterward, the suspensions were filtered through filter papers into PET bottles. The concentrations of cadmium(II) and lead(II) ions in the filtrates were determined using flame atomic absorption spectrometry (F-AAS). A relatively high initial concentration of Cd(II) and Pb(II) ions (600 mg/L) was deliberately chosen to ensure saturation of the active binding sites, enabling an accurate evaluation of the materials’ maximum loading potential.

#### 3.4.2. Effect of Temperature on the Adsorption Process

To evaluate the effect of temperature on adsorption efficiency, 0.5 g of the tested adsorbent and 50 cm^3^ of binary solutions containing both Cd(II) and Pb(II) ions at concentrations of 50, 100, 200, 600, 2000, and 5000 mg/L (for each metal) were added to 100 cm^3^ Erlenmeyer flasks. The pH of the systems was adjusted to 7 using a 1 M NaOH solution. The process was carried out using magnetic stirrers equipped with heating plates, and the flask contents were thermostated for 2 h at temperatures of 20, 40, 60, and 80 °C. After this period, the mixture was filtered through a funnel with filter paper into PET bottles. The remaining concentrations of cadmium(II) and lead(II) ions were determined using the F-AAS method.

The obtained experimental data were used to determine the parameters of the Langmuir adsorption isotherm model. To evaluate the goodness-of-fit of the experimental data to the Langmuir model, the linear coefficient of determination (R^2^) and the non-linear reduced chi-square test (x^2^/DoF) were utilized. The reduced chi-square parameter determines the discrepancy between the experimental data and the theoretical model, allowing for the verification of the hypothesized adsorption mechanism.

The Langmuir isotherm is expressed by the following equation [62]:

(1) $$q_{e}=q_{max}\cdot \frac{K_{L}\cdot C_{e}}{1+K_{L}\cdot C_{e}}$$

where q_e_ is the equilibrium adsorption capacity (mg/g), q_max_ is the maximum adsorption capacity (mg/g), K_L_ is the Langmuir adsorption equilibrium constant (dm^3^/mg), and C_e_ is the equilibrium concentration of the adsorbate in the aqueous solution (mg/L).

#### 3.4.3. Adsorption Thermodynamics

Based on the experimental data obtained from the temperature effect studies, the thermodynamic parameters of the adsorption process were determined, including the Gibbs free energy change (∆G^0^), enthalpy change (∆H^0^), and entropy change (∆S^0^). These parameters were determined using the following equations:

(2) $$\Delta G^{0}=-RT\;{lnK}^{0}\;\;$$

(3) $$lnK^{0}=-\frac{∆H^{0}}{R}\cdot \frac{1}{T}+\frac{∆S^{0}}{R}$$

ΔG^0^ = ΔH^0^ − ΔS^0^T (4)

where K_L_ is the Langmuir equilibrium constant. To satisfy the requirements of thermodynamic consistency, the original dimensional Langmuir constant (expressed in L/mg) was converted into the standard dimensionless equilibrium constant (K^0^) according to the established literature methodology [63,64]. The conversion was performed by multiplying K_L_ by the molecular weight of the respective metal ion (M, g/mol), a factor of 10^3^, and the molar concentration of pure water (55.5 mol/L), yielding a dimensionless parameter (K^0^ = K_L_ · M · 10^3^ · 55.5). R is the universal gas constant (8.314 J·mol^−1^·K^−1^), and T is the absolute temperature (K). The values of ∆H° and ∆S° were calculated from the slope and intercept of the linear plot of lnK^0^ versus 1/T, respectively.

#### 3.4.4. Adsorption Kinetics

To investigate the adsorption kinetics of cadmium(II) and lead(II) ions, 0.5 g of the tested adsorbent (bone charcoal, selected due to its superior efficiency demonstrated in the preliminary screening) was weighed into six 100 cm^3^ Erlenmeyer flasks, followed by the addition of 50 cm^3^ of a binary solution containing both Cd(II) and Pb(II) ions at a concentration of 600 mg/L for each metal. The flasks were placed on a laboratory shaker and agitated for designated time intervals: 15, 30, 60, 90, 120, and 180 min. After each specific time, the mixtures were filtered, and the residual concentrations of the analyzed ions were determined. All kinetic experiments were carried out in three independent replicates (*n* = 3), and the experimental results were expressed as the mean values. The associated measurement uncertainty was rigorously quantified by calculating confidence intervals using Student’s t-distribution for a 95% confidence level (*p* = 0.05) with 2 degrees of freedom (f = 2).

To evaluate the experimental data and predict the rate of the adsorption process, theoretical kinetic models were applied. The mathematical formulations and parameters of the pseudo-first-order (Lagergren), pseudo-second-order, Elovich, and intraparticle diffusion (Weber–Morris) models are summarized in Table 9 [65,66,67].

## 4. Conclusions

This study was focused on evaluating the application potential of volatile fly ash and bone charcoal in the removal of cadmium(II) and lead(II) ions from aqueous solutions. Based on the obtained experimental results and theoretical modeling, the following conclusions were formulated.

SEM microscopic analysis revealed significant differences in the morphology of the studied materials. Fly ash grains (356 × 374 μm) were characterized by high structural heterogeneity, whereas bone charcoal grains (570 × 560 μm) were more uniform and exhibited a tendency to form agglomerates. Energy-dispersive X-ray spectroscopy (EDS) elemental analysis confirmed the mineral-organic nature of both adsorbents. For fly ash, the dominance of the aluminosilicate phase was demonstrated, represented by high contents of oxygen, carbon, aluminum, and silicon. In contrast, the calcium-phosphate matrix predominated in the bone charcoal structure. The determined points of zero charge for both materials were similar, yielding pH_PZC_ = 8.3 for fly ash and pH_PZC_ = 8.4 for bone charcoal.

FT-IR spectroscopic studies and thermogravimetric analysis (TG/DTG/DTA) confirmed that the ion binding occurs predominantly via a physisorption mechanism involving strong electrostatic interactions and hydrogen bonding. In the case of fly ash, the active sites of the aluminosilicate matrix played a crucial role, whereas the process on bone charcoal was governed by the presence of phosphate and carbonate groups within the hydroxyapatite structure. Modifications in band intensities, shifts in their maxima, and the emergence of new signals in the FT-IR spectra provide direct evidence of the adsorption process occurrence driven by physical surface interactions.

The experimental data showed high compatibility with the non-linear Langmuir model (particularly at 20 °C, where R^2^ > 0.99). Bone charcoal proved to be a significantly more effective adsorbent for Pb(II) and Cd(II) ions, reaching maximum adsorption capacities of q_max_ = 397.55 and 325.09 mg/g, respectively, which represents a more than three-fold higher value compared to fly ash (118.22 and 105.59 mg/g). The subsequent decrease in q_max_ values with increasing temperature up to 80 °C (down to 170.59 and 141.59 mg/g for bone charcoal, and 33.55 and 32.29 mg/g for fly ash, respectively) confirms the exothermic nature of the adsorption process in the investigated system. Thermodynamic parameter analysis indicated that the metal ion adsorption process on both studied materials was spontaneous (∆G^0^ < 0, ranging from −9.55 to −19.33 kJ/mol), with a dominant contribution of physical interactions (physisorption). The negative enthalpy values (∆H^0^ = −7.27 to −14.19 kJ/mol) explicitly confirm the exothermic nature of the process. Concurrently, the positive entropy values (∆S^0^ = 5.82 to 39.09 J/(mol·K)) indicate an increase in randomness at the adsorbent–adsorbate interface during the adsorption process.

The removal kinetics of Cd(II) and Pb(II) ions on the investigated materials are best described by the pseudo-second-order model. The fitting of experimental data to the Weber–Morris intraparticle diffusion model revealed a multi-stage process, in which the key rate-limiting step is the slow diffusion of ions within the porous structure of the adsorbents. Regardless of the material type, lead(II) ions were removed faster and with higher efficiency than cadmium(II) ions, which is attributed to their smaller hydration radius and higher chemical affinity for the studied mineral matrices.

Kinetic studies demonstrated that the equilibration time varied significantly depending on the studied system. For fly ash, the adsorption equilibrium for Cd(II) and Pb(II) ions was achieved at 90 min (q_t_ ≈ 100 mg/g) and 180 min (q_t_ ≈ 118 mg/g), respectively. For bone charcoal, Cd(II) adsorption stabilized around 120 min (q_t_ ≈ 250 mg/g), whereas Pb(II) removal occurred extremely rapidly, reaching nearly 380 mg/g as early as 30 min and equilibrating at q_t_ ≈ 400 mg/g at 180 min. The pseudo-second-order kinetic model provided an excellent fit to the experimental data for bone charcoal (R^2^ = 0.997 for Cd(II) and R^2^ = 0.975 for Pb(II)), indicating that the process is controlled by a multi-step mechanism involving surface and intraparticle diffusion.

In conclusion, both low-cost mineral adsorbents exhibit high application potential in wastewater treatment, with bone charcoal being the more efficient material due to its superior kinetic parameters and higher adsorption capacity in the immobilization of heavy metals. Nevertheless, since this study was conducted under batch laboratory conditions using bi-component synthetic solutions, future research must address limitations related to real wastewater matrices, such as the presence of competing ions and organic matter. Furthermore, to evaluate the economic viability and environmental sustainability of the proposed materials, comprehensive desorption and regeneration studies are required. Testing volatile fly ash and bone charcoal in continuous flow column systems at a pilot scale will be the next crucial step toward their practical, industrial-scale implementation.

## Figures and Tables

**Table 1:** SEM-EDS analysis—quantitative elemental composition for coal fly ash (weight %).

Spectrum	C	O	Na	Mg	Al	Si	P	S	K	Ca	Ti	Fe
1	18.74	44.65	0.43	1.41	12.49	16.12	0.32	0.17	1.89	1.56	0.32	1.9
2	4.41	24.23	0.36	2.43	18.23	28.99	-	-	3.95	0.95	1.2	15.26
3	52.38	34.54	-	0.46	0.66	11.07	-	0.26	-	0.37	-	0.27
Mean	25.18	34.47	0.40	1.43	10.46	18.73	0.32	0.22	2.92	0.96	0.76	5.81
±SD	24.62	10.21	0.05	0.99	8.96	9.24	-	0.06	1.46	0.60	0.62	8.22

**Table 2:** SEM-EDS analysis—quantitative elemental composition for bone charcoal (weight %).

Spectrum	C	O	Na	Mg	Si	P	S	Cl	Ca	Fe
1	17.69	36.21	0.28	0.56	3.79	11.89	0.37	-	28.88	0.33
2	17.09	39.83	-	0.51	0.56	12.71	0.39	0.28	28.63	-
3	16.2	35.39	0.32	0.52	0.57	14.02	0.41	-	32.58	-
Mean	16.99	37.14	0.30	0.53	1.64	12.87	0.39	0.28	30.03	0.33
±SD	0.75	2.36	0.03	0.03	1.86	1.07	0.02	-	2.21	-

**Table 3:** Thermal degradation and stability parameters of the investigated adsorbents derived from TG, DTG, and DTA curves.

Adsorbent	T_5%_ (°C)	T_10%_ (°C)	T_50%_ (°C)	T_onset_ (°C)	T_max1_ (°C)	T_max2_ (°C)	Residual Mass at 600 °C (%)
Coal Fly Ash	675	-	-	550	600	675	98.1
Bone Charcoal	230	445	-	350	450	680	84.1

**Table 4:** Assignment and interpretation of the FT-IR absorption bands for the coal fly ash and bone charcoal samples [40,41,42,43].

Absorption Bands (cm^−1^)	Assignment	Interpretation
Fly Coal Ash		
3444 (3500–3000)	Stretching (–OH) and bending (H–O–H) vibrations	Water molecules adsorbed on the fly ash surface
2923 and 2854	Stretching vibrations of aliphatic CH_2_ and CH_3_ groups	Organic residues/aliphatic structures (visible after adsorption)
1636	Bending vibrations of –OH and H–O–H bonds	Hydrated mineral phases, bound water
1420	Stretching vibrations of bridge bounds C–O	Carboxyl–carbonate structures
1050	Asymmetric stretching vibrations of bridge bounds Si–O–Si and Si–O–Al	Tetrahedral silicon-oxygen and aluminum-oxygen bridges in aluminosilicate matrix
795	Symmetric stretching vibrations of Al, Si–O bonds	Crystalline quartz phase/aluminosilicates
Bone charcoal		
3420	Stretching vibrations of hydroxyl (–OH) groups	Structural –OH involved in hydrogen bonding
2013	Combination (harmonic) band	Phosphate matrix lattice vibrations
1420 and 874	Stretching (1420) and bending (874) vibrations	Carbonate groups (CO_3_^2−^) incorporated into hydroxyapatite lattice
1035 (shoulder at 1090)	Asymmetric stretching vibrations	Phosphate groups (PO_4_^3−^) of hydroxyapatite skeleton
565–602	Internal bending vibrations of O–P–O bonds	Phosphate tetrahedra (v4 vibration)

**Table 6:** Thermodynamic parameters for the adsorption of cadmium(II) and lead(II) ions onto coal fly ash and bone charcoal.

Adsorbent	Temp.		1/T	Pb(II)				Cd(II)			
				lnK^0^	ΔH^0^	ΔS^0^	ΔG^0^	lnK^0^	ΔH^0^	ΔS^0^	ΔG^0^
	[°C]	[K]	[−]	[−]	[kJ/mol]	[J/(mol‧K)]	[kJ/mol]	[−]	[J/(mol‧K)]	[kJ/mol]	[kJ/mol]
Coal fly ash (CFA)	20	293	0.0034	7.64	−7.27	39.07	−19.33	5.42	−12.89	5.82	−13.18
	40	313	0.0032	7.53				5.13			
	60	333	0.0030	7.41				5.13			
	80	353	0.0028	7.28				4.72			
Bone charcoal (BC)	20	293	0.0034	7.41	−14.19	13.30	−18.24	5.64	−9.55	14.55	−13.97
	40	313	0.0032	7.13				5.42			
	60	333	0.0030	6.94				5.42			
	80	353	0.0028	6.72				5.13			

**Table 7:** Kinetic parameters and linear regression coefficients for the adsorption of cadmium(II) and lead(II) ions onto bone charcoal.

Kinetics Model	Parameter	Bone Charcoal	
		Cd(II)	Pb(II)
Pseudo-first-order (PFO)	R^2^ [−]	0.484	0.618
	k_1_ = [1/min]	1.45 · 10^−4^	2.29 · 10^−4^
Pseudo-second-order (PSO)	R^2^ [−]	0.997	0.975
	k_2_ = [g/(min·mg)]	0.120	0.095
Film diffusion	R^2^ [−]	0.484	0.618
First stage of intraparticle diffusion	R^2^ [−]	0.825	0.825
	K′_1_ = [mg/(g·min^1/2^)]	0.247	0.247
	b [mg/g]	5.193	6.793
Second stage of intraparticle diffusion	R^2^ [−]	0.892	0.982
	k′_2_ = [mg/(g·min^1/2^)]	0.145	0.623
	b [mg/g]	6.094	2.867
Elovich	R^2^ [−]	0.970	0.789
	a = [mg/(g·min)]	123.37	33.35
	β [mg/g]	1.28	0.82

**Table 8:** Physicochemical characteristics of the investigated adsorbent materials.

	Coal Fly Ash	Bone Charcoal
Specific surface area (BET), m^2^/g	111.3	70–120
Particle size	<0.5 mm	<0.6 mm
Density, kg/m^3^	620	650
pH	9.1	7.5–8.5
Porosity (pore volume), cm^3^/g, p/p_o_ = 0.99	0.171	0.20–0.35
Chemical composition	SiO_2_ (51%), Al_2_O_3_ (20%) Fe_2_O_3_ (12.5%), CaO (4%), MgO (2%), K_2_O (0.8%), Na_2_O (0.7%)	Ca_10_(PO_4_)_6_(OH) (70–80%) CaCO_3_ (11%) Amorphous Carbon (11%)

**Table 9:** Kinetic models and their respective parameters used in the adsorption studies.

Kinetic Model	Equation	Parameters and Units
Pseudo-First-Order (PFO)	$\frac{d_{qt}}{dt}=k_{1}(q_{e}-q_{t})$ $ln\left(q_{e}-q_{t}\right)={-k}_{1}t+lnq_{e}$	q_e_—equilibrium adsorption capacity (mg/g) q_t_—adsorption capacity at time t (mg/g) t—contact time (min) k_1_—pseudo-first-order rate constant (min^−1^)
Pseudo-Second-Order (PSO)	$\frac{d_{qt}}{dt}=k_{2}{\left(q_{e}-q_{t}\right)}^{2}$ $\frac{t}{q_{t}}=\frac{1}{k_{2}q_{e}^{2}}+\frac{t}{q_{e}}$	k_2_—pseudo-second-order rate constant (g/(mg·min)
Elovich mass transfer	$\frac{d_{qt}}{dt}=\alpha e^{\left(-\beta q_{t}\right)}$ $q_{t}=\frac{1}{\beta }lnt+\frac{1}{\beta }ln(\alpha \beta )$	a—initial adsorption rate (mg/(g·min) β—desorption constant related to the extent of surface coverage and activation energy for chemisorption (g/mg)
Intraparticle Diffusion (Weber–Morris)	$q_{t}=k_{p}t^{\frac{1}{2}}+C$	k_p_—intraparticle diffusion rate constant (mg/(g·min^1/2^), t^1/2^—square root of time min^1/2^, C—constant proportional to the boundary layer thickness (mg/g).

## Data Availability

The original contributions presented in this study are included in the article. Further inquiries can be directed to the corresponding authors.
